# Understanding How Managers Deal With Mental Health at The Workplace: A Qualitative Approach

**DOI:** 10.1097/JOM.0000000000003683

**Published:** 2026-02-27

**Authors:** Elena Schwarz, Birgitta Schiller, Isabella Wagner, Kathrin Moertl, Falko Papenfuß, Michael Hoelzer, Harald Guendel

**Affiliations:** 1 From the Department of Psychosomatic Medicine and Psychotherapy, University Medical Center Ulm, Ulm, Germany (E.S., H.G.); Department of Psychotherapy Science, Sigmund Freud University Vienna, Vienna, Austria (B.S., I.W., K.M.); Sonnenberg Klinik gGmbH, Stuttgart, Germany (M.H.); and Robert Bosch GmbH, Gerlingen, Germany (E.S., F.P.).

**Keywords:** common mental disorders, stress, manager training, workplace intervention, mental health in the workplace

## Abstract

**Objective:**

More and more companies are offering specific training programs aimed at supporting managers in dealing with the mental health of their employees. Nevertheless, little is known about the individual impact factors and their effects in everyday management.

**Methods:**

Using a qualitative research approach, 20 managers were asked about their experiences with a training program after 1 and 12 months.

**Results:**

Analysis showed an overall great interest and open attitude among the participants. Still, ambivalences emerged when it came to implementing the training and actually dealing with mental health problems. Typifying the participants showed different stages to describe the managers’ individual attitudes concerning mental health at the workplace.

**Conclusion:**

The results could hold great potential for more targeted individualization of manager training courses in the field of mental health. However, further research is needed.

## Abstract 2

Managers’ level of knowledge and willingness to engage with mental health at the workplace varies widely. The results of the present study can help to develop holistic interventions and training approaches for companies and their managers to actively shape mental health in everyday working life.

## Graphical abstract

**Figure:**
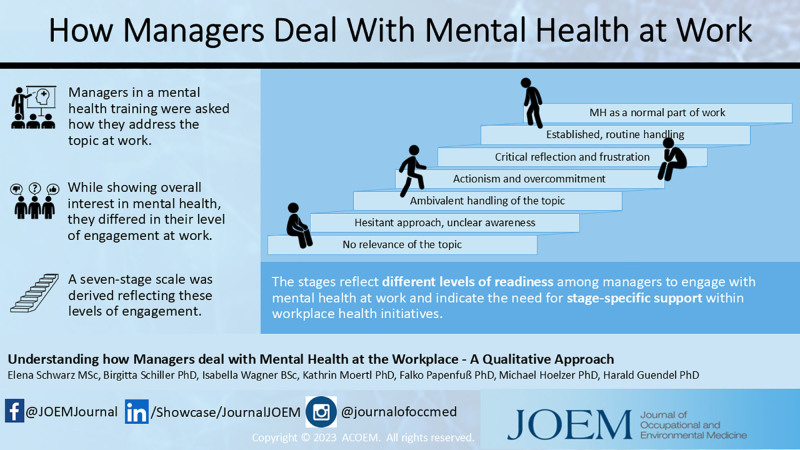

**LEARNING OUTCOMES**

- The responsibility to recognize and address mental health risks or changes within the team puts many managers under pressure.
- In terms of understanding and dealing with mental health in the team, managers are at different levels of processing that should be considered as part of holistic health interventions.

In recent years, the prevalence and consequences of common mental disorders, such as depression and anxiety, have become increasingly important in companies.^1,2^ On the one hand, poor employee health conditions may cause high economic costs by leading to sick leave, working with reduced productivity (absenteeism), and increased staff turnover.^3–6^ On the other hand, the workplace is a possible risk—but also a protection factor^7^ and thus brings employers to consider possible adjustments. For example, in Germany, there has been a steady increase in work demands and subjectively perceived work stress.^8^ In addition to offers for physical health, interventions that address the mental health of the workforce are therefore increasingly in demand.^9^

There is already a variety of guidelines and interventions^10^ developed by many different fields of expertise.^10,11^ Thereby, approaches comprise preventive, intervening, and rehabilitating strategies.^12–15^ Also, interventions differ in terms of the target group (eg, employees, managers, and companies), in terms of their focus (eg, providing information, teaching techniques...) and also in terms of the methodology (online, on-site, individual, in a group...).^12^ Even if not all interventions find their way into a scientific evaluation, small to moderate effects can be shown in the literature for many approaches.^16^ Nevertheless, stress-related illnesses, and thus common mental disorders, are still on the rise.^17^ Therefore, it seems crucial for public health care as well as companies to expand approaches on stress reduction and mental health promotion at the workplace.

Thinking about adequate implementation of health campaigns, managers are taking on a big part in the process. Since the end of 2013, the German Occupational Safety and Health Act has explicitly required that psychological stress be considered in the regular risk assessment. In the companies, the responsibility for the realization falls to the managers. In addition to this important control function, they may also have a direct influence on the health of employees, including mental health.^18^ Studies showed that the leadership style, the quality of the relationship, and task orientation, as well as the quality of the supervisor-employee interaction, have an impact on the mental health of employees. On the other side, managers are exposed to a lot of stress themselves, especially in the middle management level. The risk for employees in management positions of developing a stress-related illness, such as cardiovascular diseases,^19^ coronary heart diseases,^20^ depression,^21^ or burnout is increased.^22^ In addition, a large amount of work stress can, in turn, lead to managers passing it on to their employees.^23–25^ The management style and the design of working conditions can also be negatively influenced by a high level of stress.^26^ Thus, stressed executives tend to fall back on destructive leadership styles and neglect the creation of healthy working conditions. A further investigation showed that the influence of leadership on the stress experience of employees was mediated by mental depletion.^27^ Enabling managers to better deal with their own stress level and those of their employees, therefore, seems to be central.

From 2016 to 2018, an approach was applied in a large German company that aimed at training managers in assessing psychological hazards in the workplace and in dealing with stressed employees.^28,29^ The quantitative evaluation showed an improvement in the mental health of the participating managers after 3 months. Also, the 6-month follow-up showed an improvement in attitudes towards mental illness among the participants. However, as being part of a field research survey, it was not possible to make reliable statements about the exact mode of action of the approach. Recommendations for further realization can therefore only be formulated to a limited extent.

Thus, to gain an insight into the implementation processes and about what managers are planning to realize in their department after attending the 1-day seminar, a qualitative survey of the participants was conducted additionally. For this purpose, individual participants were asked about their experiences with the training and their attitudes towards mental health in the company.

## METHODS AND MATERIALS

### Sample

**FIGURE 1.**
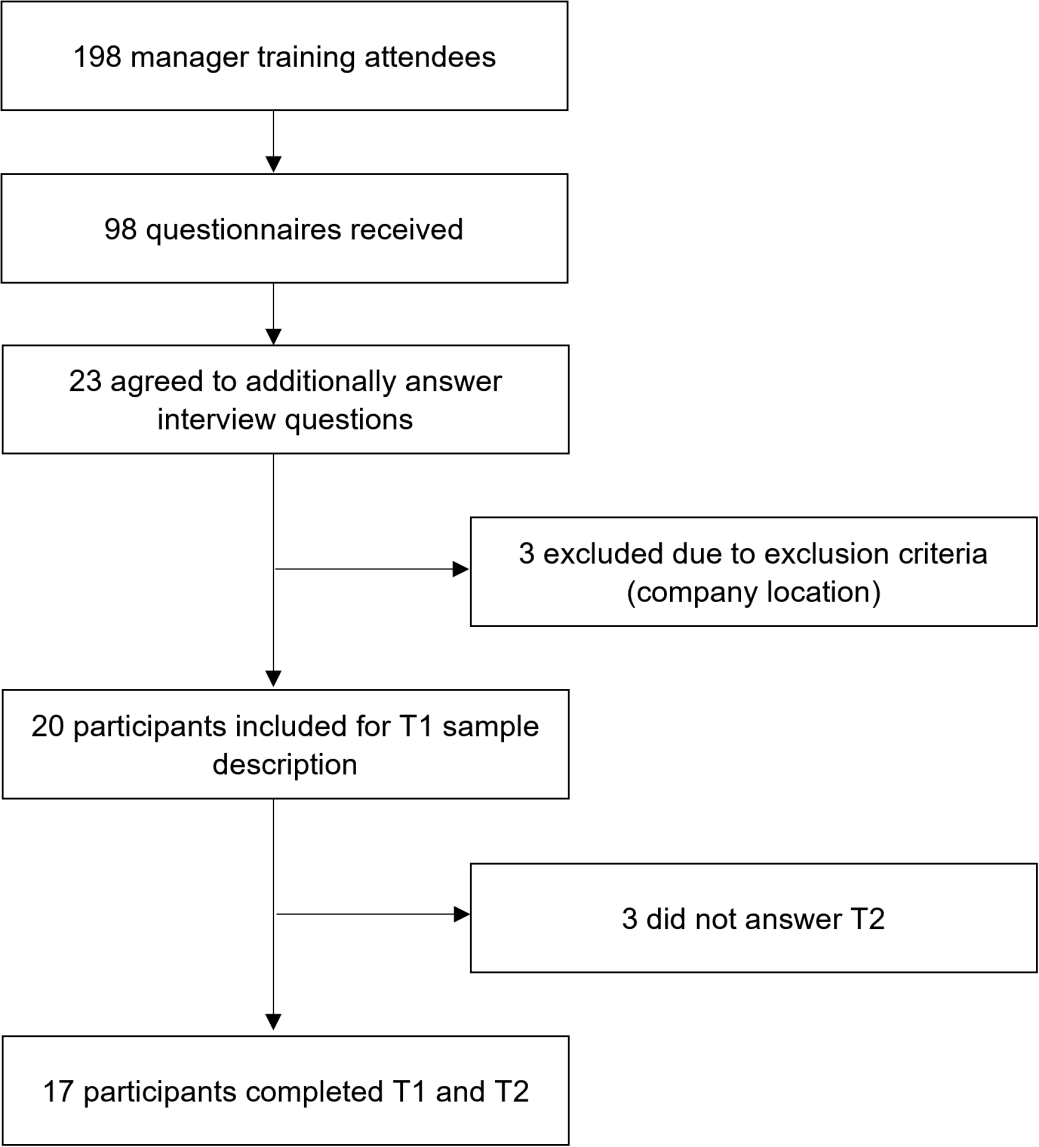
Selection process dataset.

The data presented in the current article were collected from n = 20 managers working for a large industrial company in Germany. All participants took part in a specific manager training addressing mental health at the workplace in between October 2016 and August 2018. The trainings were provided at 3 locations of the company: housing headquarters, research and development, as well as purchasing and logistics. Purposeful sampling was applied by recruiting managers from the quantitative evaluation of the training for participation in the qualitative survey. Out of N = 198 training participants in total, n = 98 (49.5%) filled in the quantitative questionnaires. Another n = 23 (11.6% of the total) agreed to answer the interview questions. Three had to be excluded, as they were not working in 1 of the 3 company locations included in the survey. Thus, n = 20 managers participated in the first interview wave. During the ongoing analysis, data saturation was meticulously assessed. No new first-level codes, categories, or process-related patterns emerged in the later interviews, indicating data saturation at both the categorical and process level. The final interviews primarily confirmed and further differentiated previously identified patterns rather than generating novel insights. At the 12-month follow-up, n = 17 participants still could be reached for the second measurement point, which further supported the stability and saturation of the identified process model. Figure 1 illustrates the recruitment process.

### Manager Training

The training took place at the company’s site and was implemented in collaboration with the Department of Psychosomatic Medicine and Psychotherapy of the University Medical Center in Ulm and the Sonnenberg Klinik gGmbH in Stuttgart. Managers were able to voluntarily sign up for 1 of the single-day workshops within the general training program on the company’s intranet. All workshops were held in seminar rooms equipped with both a computer and a projector. Each group consisted of a maximum of 15 participants. The content structure of the intervention has already been published in detail elsewhere.^29^ Nevertheless, a brief overview is given at this point for the sake of readability.

The training day was divided into 3 parts with 3 different goals. First of all, the participants should be informed about their tasks in the field of psychological risk assessment. This included information on the legal framework and practical implementation, as well as information on important contacts in the company. The second part of the day aimed at training participants in how to perceive their own stress symptoms and those of their employees. This included teaching basic knowledge about important models in the field of work stress. Typical stress and stress symptoms were also worked out together, and strategies for dealing with stressful work situations were discussed. This happened with the active integration of the personal experiences of the participants. The last part of the training consisted of case discussions. Participants were asked to present difficult situations they experienced in everyday work. Those were discussed in the group, guided by the trainer.

### Qualitative Instrument

To examine the managers’ attitudes concerning mental health at the workplace, semi-structured interviews were created for 2 measurement points. Interviews were conducted in between 4 weeks as well as 12-month after the manager training. Each time, the participants were asked to describe their position within the company as well as change requests concerning their work routine. Also, they were asked to outline their self-perception as a leader. In addition, managers were asked about their motivation to attend the offered training (eg, “How would you feel talking about mental health and stress management to your employees?”). Changes observed in the company concerning the addressed topic were examined as well (eg, “Did you observe any changes in your leadership due to the project and the manager training?”). Finally, we asked the participants to describe the usual way to deal with mental health problems within the company. Furthermore, we asked participants to draw up proposals for improvement. The interview guidelines can be accessed at Supplemental Digital Content 1, https://links.lww.com/JOM/C390, freely translated from German.

### Quantitative Instruments

In addition to the qualitative interviews, participants were asked to fill in several validated questionnaires at 3 different times of the survey (before the training, as well as 3 and 12 months afterwards). The questionnaires addressed the participant’s knowledge and attitudes concerning mental health. Also, managers were asked about the state of their own physical and mental health, as well as their current working situation. The detailed analyses of the quantitative data were published elsewhere.^28,29^

### Implementation

A few days in advance, the training participants received an email to get informed about the associated study. The email also included the quantitative questionnaires. On the training day, one of the trainers informed us again about the possibility to participate in the survey. Questionnaires as well as written consent forms were distributed. At the end of the questionnaire, participants were asked to sign up for the additional interviews. Those who agreed were contacted by one of our researchers (E.S.) a few days after the training. Personal interviews were held by one and the same researcher at the company site. All first measurement point interviews were conducted within 4 weeks after the manager had participated in the training. The data was audiotaped. Twelve months after the training, the managers were contacted for the second time. Again, personal interviews were held at the company side by one and the same researcher and audiotaped for further processing.

### Design and Data Analysis

This qualitative study employed a naturalistic design and was conducted and reported in accordance with the Standards for Reporting Qualitative Research guidelines.^30^ All items on the Standards for Reporting Qualitative Research checklist were addressed and are available in Supplemental Digital Content 2, https://links.lww.com/JOM/C391. The data were processed using ATLAS.ti 7^31^ and analyzed iteratively throughout the study. Thus, material from the first measuring point was analyzed first. Interviews at t_1_ lead to about 570 minutes of audio data material. After 12 months, another 484 minutes of data material were collected at t_2_.

Data analysis was performed by 2 independent evaluators based on the grounded theory. Both were not involved in conducting the interviews. In the beginning, first-level codes were generated simultaneously by both evaluators. A parallelization took place afterwards. In total, 894 first-level codes were defined. In a second step, first-level codes were categorized into second-level codes. This led to 56-level codes. At the second measurement point, the categories from the first analysis were used for coding. Only unknown information was given new codes. To ensure qualitative validity, the analyzing process was supervised on a regular basis by a scientific working group.

Following the inductive open coding, axial coding was conducted to explore relationships between categories during this phase; different patterns in managers’ approaches to mental health at work became apparent. To specify these types, we followed the guidelines of Kluge^32^ on systematic and comprehensible typologization. However, the analysis revealed that managers could not be meaningfully classified into static types. Instead, the data indicated different levels of processing and engagement with the topic of mental health, which appeared to be dynamic and potentially sequential in nature.

A process model was conceptualized when considering these results while gaining further information of t2. In a subsequent analytical step, this model was further refined through abductive reasoning by relating the empirically derived process levels to existing process models from the therapeutic field. Thereby, we identified a close resemblance between our empirically derived process levels and the Heidelberg Restructuring Scale (HUS), which subsequently served as a sensitizing theoretical framework to further specify and contextualize the findings. This abductive linkage did not guide the initial inductive coding process but was applied after the analysis to enhance theoretical coherence and interpretability; the HUS is therefore outlined below.

#### The Heidelberg Restructuring Scale

The HUS is a model describing the change or restructuring of the patient’s personality in psychoanalytic therapies and operationalizes them using an assessment scale.^33^ HUS is further developed on the basis of the Assimilation of Problematic Experiences Scale.^34^ To assess the therapeutic development within a certain area, the scale describes 7 levels, each having 3 different characteristics. Level 1 comprises patients not yet being aware of the focus topic. Psychodynamically, it is assumed that for defense purposes, the patient unconsciously avoids coming into cognitive or emotional contact with the conflict issues. At level 2, there is an unwanted occupation with the focus area, for example, due to disruptive effects on the patient’s everyday life. At this point, dealing with the problems is most likely forced by external pressure. In stage 3, the patient’s perception of the problem intensifies and goes along with discovering possible intrinsic parts of the problem. At this stage, the patient also examines the topic in depth, primarily caused by external pressure or internal tension. Patients at level 4 are already actively and aggressively dealing with the focus topic. They begin to take on responsibility and experience their own freedom of action in dealing. Changes arise through the conscious cooperation of the patient. In stage 5, the patient is overwhelmed by the focus problem. This means that he no longer actively deals with it, as in stage 4, but was forced to do so. He realizes that the focus topic is part of himself. The patients perception and affective attitude towards the outside world and other people become increasingly more realistic. At level 6, there is an acceptance of existing circumstances and deficits, and relief occurs. Finding a solution in dealing with the focus problem no longer takes place actively and with effort, as in level 4, it runs passively. Patients at level 7 have certainly acquired changes. Gratitude for the past arises, and dealing with the focus problem can be experienced in a balanced way.

The subsequent abductive linkage to the HUS served as a sensitizing theoretical framework to further specify and contextualize the empirically derived process levels. This step did not guide the initial coding but was applied after the inductive analysis to enhance theoretical coherence and interpretability.

### Reflection on the Researcher’s Perspective

As part of the semi-structured interview technique, follow-up questions were regularly asked. Although these were formulated openly (eg, “Would you please elaborate?”), the decision regarding whether to ask a follow-up question was made by the interviewer. Although the guiding questions ensured that the interviews were largely comparable, confirmation bias cannot be completely excluded during data collection.

### Ethical Approval and Registration

This survey was conducted in accordance with the Declaration of Helsinki, and the study protocol was approved by the Ethics Committee of Ulm University (326/16). The investigation was registered by the German Clinical Trial Register (DRKS) under ID number DRKS00011371.

## RESULTS

### Participants

N = 20 managers were interviewed at the first measuring point. All of them were male. The age ranged between 36 and 59 years (M = 47.9, SD = 6.8). Overall, n = 19 were living in a relationship. Most of the participants had between 6 and 15 employees working in their team. Four had only up to 5 employees in the team, and another 4 supervised more than 15 employees. Seven participants worked in the research and development department of the company, 7 in purchase and logistics department, and the remaining 6 in the controlling section. The positions of the managers ranged from team leader (the lowest) to head of department (second highest) within the company. Table 1 shows the baseline sociodemographic information of the interview participants compared with all participants within the 3 locations.

**TABLE 1.** Sociodemographic Information of the Interview Participants Compared With the Total Sample at the 3 Investigated Locations

	Interview Participants N = 20	Total Sample N = 57
Gender	n = 100 male (100%) n = 0 female (0%)	n = 50 male (87.7%) n = 7 female (12.3%)
Age (years)	M = 47.9; SD = 6.8	M = 47.1; SD = 8.3
Relationship	n = 19 (95%)	n = 53 (93%)
Children	0: n = 3 (15%) 1: n = 3 (15%) 2: n = 11 (55%) 3: n = 3 (15%) 4: n = 0 (0%)	0: n = 9 (16.1%) 1: n = 10 (17.9%) 2: n = 24 (42.9%) 3: n = 11 (19.6%) 4: n = 2 (3.6%)
Secondary education	Lower: n =1 (5.3%) Middle: n = 0 (0%) Higher: n18 (94.7%) Others: n = 0 (0%)	Lower: N =1 (1.8%) Middle: N = 3 (5.5%) Higher: N = 50 (90.9%) Others: N = 1 (1.8%)
Position	Team leader: n = 4 (20%) Group manager: n = 7 (35%) Department manager: n = 4 (20%) Head of department: n = 2 (10%) Others: n = 3 (15%)	Team leader: n = 4 (7.1%) Group manager: n = 22 (39.3%) Department manager: n = 15 (26.8%) Head of department: n = 3 (5.4%) Others: n = 12 (21.4%)
Employees supervized	1–20: n = 16 (80%) 21–100: n = 3 (15%) >100: n = 1 (5%)	1–20: n = 42 (76%) 21–100: n = 12 (22%) >100: n = 1 (2%)

M, mean; N, number; SD, standard deviation.

### Categories

Within the first coding procedure, 894 codes were identified for the first measurement point. These first-level codes were then categorized into 56 second-level codes. At the second measurement point, another 219 codes were identified and added to the already existing second-level codes of t_1_. The evolved 275 codes were then conclusively categorized into third-level codes, which, in this case, outline a process model. The final codes will be outlined in detail in the following subsections.

#### Company Culture Versus Modern Work Culture and “Work Without Boundaries”

Company culture is very people-oriented. People are valued for their ability to be intelligent, and therefore, it is important to take care of the employees. In this context, it is understandable that leadership culture has a similar orientation. The managers describe themselves as open, team-oriented, communicative, and flexible.

On the other hand, there are developments within the modern work culture that put pressure on employees, especially on those in leading positions. Expectations to work more freely and creatively are described in parallel with even more limiting guidelines. New technologies create new areas of work, but sooner or later, employees with different skills will either have to be retrained or dismissed. This represents an emotional dilemma for managers who (which is also required in modern work culture) appreciate and are emotionally bound to their employees. Also, it is hard to cope with a flood of information, and rigid processes make innovative things impossible.

Even though there are many and varied interventions aiming at strengthening mental health (yoga, stress management training, communication seminars, etc.) in accordance with the “People are the most important good” company ideology, framework conditions do not ensure mental health stability. Increased mental health knowledge does not automatically help when experiencing deteriorated working conditions.

#### Mental Health and Its Inhibitions as well as Stigmas

In addition to social values, mental health is a basic pillar in both company and work culture. All executives confirm its importance as it concerns a high proportion of employees. Interestingly, asking participants about existing working groups, some give avoidant answers such as: “No, there is no need for us”; “Hopefully we don’t need that”; “I don’t know what this is supposed to do, there haven’t been any problems so far”; “We are so well positioned that we do not need it, maybe other companies without social values.” On the other hand, other participants stated that implementing a good mental health practice at the workplace needed time. Also, the implementation process not only depends on the acceptance by the companies but the society in general.

It was also explained that there are still inhibitions, although there is a high level of understanding in the company. These are mainly based on existing stigmatizations. Physical impairment, for example, is accepted as an illness. Mental illnesses are still considered a weakness caused by the person themselves (eg, weak self-control and lack of stability). Thus, the mental health concept is more associated with one’s own “inability” than an approach to a healthy and desirable lifestyle. Considering this fact, it makes it difficult for managers to talk with their employees, because they do not want to cross borders or give them the feeling of not being good enough.

#### Expectation of Mental Health and the Professional Limit

Leading a company respecting the new mental health guidelines makes managers feel responsible for ensuring its implementation. As mental health can be influenced by various factors, this task seems impossible for managers not being trained in the psychological field. Still, the interviewees faced this challenge in different ways. Some stated that there are employees who do not want any help. Statements such as “they have to come by themselves” or “this does not help in severe cases” indicate a certain helplessness. Indirectly, managers were afraid of being accused of having a “work area that makes sick,” for example, because of difficult team dynamics or “bad leadership.” However, others appeared very self-confident and able to deal with mostly everything. They did not feel under pressure or in complete responsibility, but rather took on the perspective of the employees. The main difference between both views is that those who seem to be able to react more calmly already had experiences with or were affected themselves by mental health issues.

Clear differences regarding the qualification were also found. Many executives seemed overwhelmed by the new challenge: “It’s like driving a car without a driver’s license.” Managers who could rely on past experiences were much more relaxed. They relied on their employees to seek for talk if needed and created a trustful atmosphere to address difficult subjects.

#### Perplexity and Frustration Versus Awareness Takes Time

As already stated, the noticeable ambivalence in the company was also reflected by the participating managers. On the one hand, there was a clear resistance; on the other hand, a very high level of effort. For those with a reluctant attitude, topics of modern work culture were much more in the foreground. It can be assumed that, at this time, this issue was more important and therefore did not leave any capacity for additional or expanded tasks and responsibilities. Those who were very engaged in the mental health topic tried very hard and sometimes even got frustrated. One reason for frustration was, for example, the unmanageable variety of offers. There has also been frustration about the fact that only those who were already interested made use of the offers. Others, who might probably need them even more, were not reached.

Another group of interviewees seemed to have put all the frustration and anger aside. In no way did they show a passive attitude, but rather a calm urgency. They were aware of the problems, and they also addressed them very clearly. Nevertheless, they also pointed out that changes need time. According to them, the issue of mental health needs to be raised in society in general. These managers were also no longer concerned about the new challenges of modern work culture. They showed tremendous confidence. Even though they were not happy with the current conditions, they seemed to have arranged with them. Mental health is an attitude towards life and not a program that can be initiated, and once it is achieved, it requires active and permanent awareness to become normal.

#### Training and Implementation of Mental Health and Suggestions for Improvement

All managers experienced the training as positive and enriching. The practical expert input was particularly helpful and enhancing. Some topics only gained awareness and became clear through the training. Attendance was voluntary. Senior leadership expressed concrete interest, which helped to take advantage of the offer. Though employees had not been informed about the training. During the interview, 1 manager noticed the discrepancy and wrote himself a memo to inform the employees. There were no changes perceived in the working processes due to the implementation. Yet, some managers noted a more open discourse within the company and that more people were now talking about mental health. Especially the exchange with colleagues in comparable situations was perceived as very helpful during the training days.

Some of the managers noted the necessity of positive wording while talking about mental health to avoid taboos and to signal willingness to communicate. Communication in the company should be strengthened and structured to prevent employees from getting lost in a flood of information.

### Conceptualization

**FIGURE 2.**
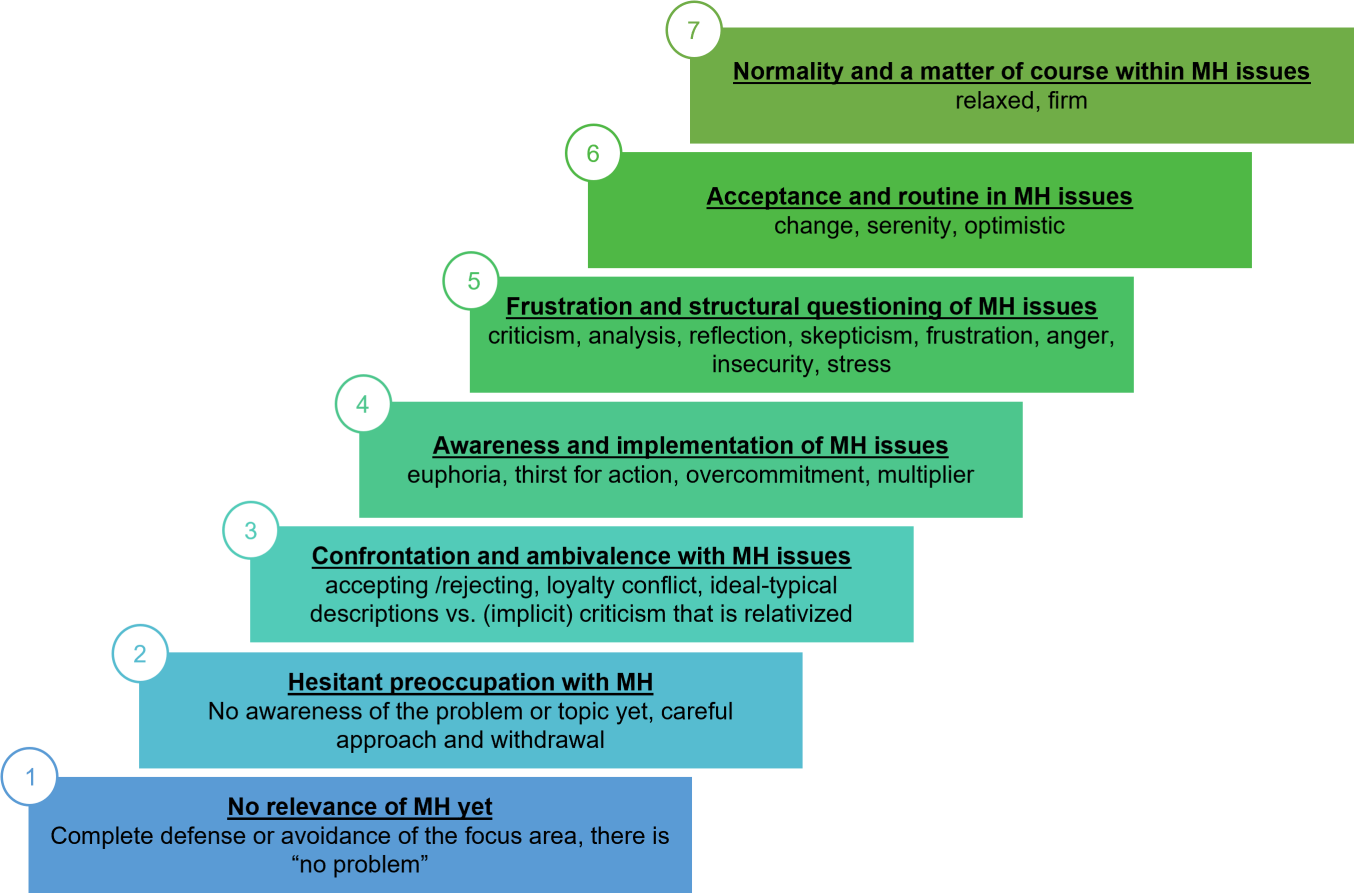
Process stages within the operational continuum of implementing MH at the workplace. MH, mental health.

Based on the analysis presented above, a typologization of the participants was performed after the first measurement point. Within this typologization, two ideal types were classified: type 1 “in process” and type 2 “in slow process.” Leaders belonging to type 1 stated mental health at the workplace to be a very important issue. In the meantime, they worried about crossing professional boundaries (e.g., “How far can I go?,” “You don’t want to make everything worse,” “It’s like driving a car without a license”). Leaders belonging to type 2 also stated mental health at the workplace to be a very important issue, but already learned that acceptance and awareness take time (eg, “The first step is done, the rest will find its way,” “Now it has to grow, that takes time”). However, it appeared that the participants were at different stages of processing the focus topic. This suggested a process-based development of one’s own attitude towards mental health in the workplace. Stages could be outlined by comparing the 2 types with the material from the second measurement point. This also represents the core code and is therefore the main result of the qualitative analysis of the executive interviews. The process stages generated are outlined below and shown in Figure 2.

### Core Code: The Operational Continuum of Implementing Mental Health at the Workplace

#### Level 1: No Relevance of Mental Health Yet

Level 1 comprises managers who completely avoid mental health issues and state that there is “no problem.” Level 1 thus refers to those managers who neither came to the training nor registered for an interview in the first time. These cases are only reflected in the narratives of the interviews. Executives who do not yet consider the topic of mental health to be relevant are not included here.

#### Level 2: Hesitant Preoccupation with Mental Health

At this level, there is no problem awareness and no conscious handling of the mental health topic, but rather a passive preoccupation. There is a careful approach and withdrawal. There were contradicting and irritating statements. On the one hand, ideal-typical descriptions were chosen that correspond to a desired image of a capable manager. Mental health is seen as an essential and important factor; its relevance is recognized, and it is known how important it is. On the other hand, there are no difficulties perceived in the own team or company, and there is no need for changes or support.

#### Level 3: Confrontation and Ambivalence with Mental Health Issues

In stage 3, participants face a loyalty conflict. Managers criticize processes but relativize their view in the next moment. Typical statements were “yes, but the company is already doing so much” or “that can be explained by the size, the company is not responsible for that.” It seems as if difficulties cannot yet be fully expressed and need to be diminished. The inner image of good work must not be destroyed.

#### Level 4: Awareness and Implementation of Mental Health Issues

At level 4, it is already possible to actively deal with the topic. Interviews showed that managers now have a completely different view and that there was a moment when everything came clear to them. Managers in this phase are energetic, interested, and optimistic about changing something. That makes them good multipliers. There is a lot of goodwill up to euphoria and determination. Mental health is approached very actively and consciously. Sometimes there is overcommitment. The integration and opening up is new, but what is still missing is the absolute clarity about the entire dimension of the consequence that the implementation of mental health brings about.

#### Level 5: Frustration and Structural Questioning of Mental Health Issues

In stage 5, a feeling of existential exposure arises. Since the managers previously participated very actively in processes and activities, they now recognize that, despite great efforts, the issue cannot be resolved by simply addressing it. Misunderstandings, perplexity, frustration, and anger spread. The perception of doing everything but not being able to change the world leads to exhaustion and triggers a crisis.

These are the managers who do everything, and yet their employees suffer from burnout. Initiatives ranging from yoga to stress management interventions do not seem to ease the pressure. Those are the executives who realize that there is something wrong with the system and demand for more action than just talking. Despite the large amount of critique at this level, it is important to take the managers seriously and enable discussions.

#### Level 6: Acceptance and Routine in Mental Health Issues

At level 6, managers have found their way out. They seize courage and are reconciled. They can accept and tolerate difficulties more easily and no longer blame anyone. Existing deficits can be clearly addressed. In contrast to level 4, this happens unintentionally, and it seems as if a common understanding has developed and is automatically applied.

#### Level 7: Normality and a Matter of Course Within Mental Health Issues

A complete integration of mental health at the workplace has been achieved. The executives assume their full (possible and appropriate) responsibility. They know about their responsibilities and limits. Also, they are able to recognize the responsibilities and limits of others (superiors and employees, society and state). Executives at this level seem to be self-assured, very determined, and not easily upset. They understand the ambivalence of the world, and they know about their own influence potential. However, the topics are not actively addressed, but whenever necessary. Dealing with mental health has become quite normal—mental health being rather an attitude towards life than a program.

## DISCUSSION

In the present study, managers were asked about their attitude towards mental health at the workplace at 2 different times after a specific intervention. When evaluating the material, it became clear that ambivalences arose in both groups on an individual level. Mental health plays an important role in the company, and the employer puts people first, but modern work culture (eg, working from home) and potential work overload are often opposed to this.^35–37^ Although interviewees also regard mental health as important, they report stigma and inhibitions on both the managers’ and their employees’ side. When it comes to psychological risk assessment and management, in particular, the requirements for managers are high. Managers feel insufficiently qualified in this field and reach their limits. Some participants were frustrated and quite perplexed, while others were sure that raising awareness simply needed more time. Different stages were conceptualized to describe the managers’ individual attitudes concerning mental health at the workplace. Thus, the main finding of this qualitative investigation is that the surveyed managers are at different levels when it comes to dealing with mental health at the workplace and accordingly need different support in dealing with emerging challenges.

This knowledge could be of great importance in the design of future workplace interventions. Assuming that the employees of a company are all at different stages of approaching healthy working conditions, it suggests that different interventions are needed as part of an effective health initiative. Thus, while the selection of individual offers may be of great benefit to groups of employees, it is likely to be difficult to apply to an entire company. Given the wide range of interventions and differences in their effectiveness, it can be challenging for organizations to put together an appropriate offer for their employees.^38^ In addition, it has been shown that successful health management should remain flexible and adapt to the needs of the company.^39^ The defined stages can provide structural support for holistic interventions and guide the selection of appropriate modules. For example, employees at the lower levels could benefit from introductory events or health days that carefully bring up the subject in everyday working life. Themed workshops or training courses can address employees at the middle levels by presenting more information and skills for those who are already interested. On the other hand, employees at the upper levels might benefit from regular focus groups for exchange and further practice. It already has been shown that one challenge in offering interventions for a group of people is the different previous experiences and needs of the participants.^40^ The extent of learning and knowledge transfer can be positively influenced by a previous needs analysis.^41^ Thus, further research on the model presented could support a better assessment of companies and thus help developing customized large-scale health offensives.

Another hypothesis posed by this model is that the willingness of managers to engage with the subject of mental health is not necessarily a conscious decision, but rather an internal process of approach and defense. This process is influenced by other topics that are currently more or less prioritized, such as upcoming tasks or the introduction of new work processes. Thus, managers who need to focus more on other topics could perceive new tasks as additional pressure in their everyday work. In fact, this could reduce their willingness and ability to deal with them. Managers who are not yet ready to deal with the issue of mental health can, in return, also exert pressure on other important actors. For example, company physicians or personnel managers who help to design and implement appropriate training courses. The therapeutic perspective^42^ of the presented model could be helpful in relieving the pressure on everyone involved.

Overall, it was shown that the surveyed participants regarded mental health at the workplace as a relevant topic in today’s working world. They also stated that, in a management position, they felt responsible for the mental health of their employees. This result is, of course, encouraging, given the various forms of stigmatization that still exist within the working world^43^ and the influence that leadership behavior can have on the mental health of employees. On the other hand, participants reported that the feeling of being responsible can also turn into a major stress factor. In some cases, an employee’s illness is directly linked with poor leadership. It seems that it is not enough to inform and train managers; successful implementation also appears to be influenced by the working environment and the corporate culture. In times when mental health becomes a focus topic in many companies, it needs a culture of discussion that allows an open discourse about stresses and uncertainties. A construct that has been frequently discussed in this context is psychological safety. It describes an individual’s perception of being safe when taking interpersonal risks.^44,45^ Especially, the model of psychosocial safety climate has often been the subject of occupational research in recent years.^46^ It refers to organizational guidelines, practices, and procedures within the company that aim to protect the employees’ psychological health. Research showed that the employees’ perception of psychosocial safety predicted changes in engagement, reduced job strain, and had positive effects on mental health.^46,47^ Especially in times of change, psychological safety seems to be a relevant factor.^48^ Companies that want to support their managers and employees in developing a healthy working attitude should take this into account in their organizational guidelines, practices, and procedures.

It can be said that the study holds great potential for more targeted individualization of workplace interventions in the field of mental health. However, the interpretation of the results should be understood as exploratory for the development of a hypothesis for further research. Process models are already known and established, especially from psychotherapy research.^33,34,49^ These all relate to the individual level in the therapeutic contact and are certainly not easily transferable to a whole company. Nevertheless, we consider the observations presented as an important first step to better understand the complex processes involved in the implementation of health offensives in companies. The further development of the model could make it possible to better adapt interventions to the different attitudes of the target group. More studies comprising both qualitative and quantitative approaches will be needed.

### Limitations

A clear limitation of the study was that our sample only consisted of male managers. This was certainly influenced by the higher proportion of male managers in the companies’ locations that were included in our survey. Still, the lack of nonmale participants limits the generalizability of the data.

Another limitation that needs to be discussed is the use of the HUS within the conceptualization process. We analyzed the material exploratively according to the grounded theory and thus conceptualized a process model. The further development included a literature research on existing process models that brought up the HUS as a very similar approach. Therefore, it must be assumed that the final formulation of the process stages was influenced by the formulations of the stages within HUS.

## CONCLUSION

The current survey attempted to better understand managers in dealing with mental health at the workplace after a specific training. Whereas people were mostly interested in and openminded towards the topic, the survey showed that the responsibility associated with this at the same time posed a large burden. Considering the attitude as a circulating process on different levels of development could help to better adapt interventions and prevent unhealthy overload. Still, this interpretation is based on one company and only male managers. Clearly, more focused and more detailed research is needed to transfer the findings on a next level.

## Supplementary Material
