# Supplementary material for: Understanding How Managers Deal With Mental Health at The Workplace: A Qualitative Approach
Source: J Occup Environ Med. 2026 Feb 27;68(8):646–54. doi: 10.1097/JOM.0000000000003683 (PMC13361957; doi:10.1097/JOM.0000000000003683)
Supplement: Supplementary file 1 [file joem-68-646-s001.pdf]

Supplementary Digital content 1: Manager Interview

INTRODUCTION: WORKING POSITION AND THE COMPANY IN GENERAL

- How would you briefly describe yourself as a manager in two to three sentences?
- If you could change anything about yourself what would it be?
- If you could change anything about the work / company / employees what would it be?

TRAINING: MOTIVATION AND ACCESS

A couple of days ago you attended the training…

- How useful or necessary was it for you?
- From your point of view, which employees need support in form of a care talk or psychosomatic consultation and which do not? Why and why not?
- How open are your employees concerning the topic?

FIRST EFFECTS

Has anything changed in the company since the trainings started…

- Has anything changed for you as a manager due to the project or training?
- Has anything changed for the employees as a result of the offer?
- Since then, has anything changed in the company or in the working atmosphere?
- Is there anything difficult or hindering you in this context? What do you think is difficult for your employees?

WORK ENVIRONMENT

If you think of the work environment and your employees, but also maybe of colleagues or higher-level superiors, ...

- How does your company deal with stress and mental health?
- How are these topics communicated in the company?
- Do you have any suggestions on how the entire project and its' organization could be improved?

IS THERE ANYTHING ELSE YOU WANT TO TELL?
